# Adolescent Risk Behaviours and Family Settings in Bulgaria: An Evidence-based Approach to Effective Family Support Policies

**DOI:** 10.34763/jmotherandchild.20212503SI.d-21-00013

**Published:** 2022-04-30

**Authors:** Elitsa Dimitrova, Tatyana Kotzeva

**Affiliations:** 1 Institute for Population and Human Studies – Bulgarian Academy of Sciences & Plovdiv University Paisii Hilendarski Plovdiv Bulgaria; 2 Burgas Free University & Institute for Population and Human Studies – Bulgarian Academy of Sciences Burgas Bulgaria

**Keywords:** Family setting, interaction of family affluence and family support, cigarette smoking, alcohol consumption, cannabis use, sexual debut, parental component in health promotion programs

## Abstract

**Background:**

The paper focuses on Bulgarian adolescents’ behaviours that put their health at risk and their relationship to family-related characteristics: structure of family and material status, family support, communication with parents, parental monitoring and school-related parental support. It also discusses intervention programs with a focus on parent support gradient.

**Material and methods:**

The analysis is based on the Bulgarian sample of Health Behaviour in School-Aged Children survey, the 2018 round. Logistic regression models of current cigarette smoking, regular alcohol consumption, sexual debut and current cannabis use are applied. Main national programs on adolescent health and the parental involvement component in them are also discussed.

**Results:**

The statistical analyses reveal significant gender and age differences in Bulgarian adolescents’ health risk behaviours. Girls have significantly higher odds of smoking and are less likely to report an early start of sexual life. Odds of cigarette smoking and regular alcohol consumption increase with age. Children living with one parent have significantly higher odds of smoking, regular alcohol consumption and current cannabis use. Interactions between FAS and family support reveal that students who report low family support, regardless of the material status of the family, have significantly higher odds of health risk behaviours.

**Conclusion:**

The main contribution of the analysis reveals the alleviating effect of family support on socio-economic inequalities between families. An evidence-based approach delineating a preventive potential of family support on Bulgarian adolescents’ health risk behaviours despite the level of family affluence provides solid arguments for increasing national family support programs.

## Introduction

Adolescence has been recognised as a period of emotional intensity, cognitive explorations, novelty- and experimentation-seeking, part of which could entail health risk-taking behaviours. Jassor’s theory on problem-behaviour outlines the importance of the interaction between adolescents and their environment [1]. This theory has been applied in the domain of adolescent health-compromising and health-enhancing behaviours, revealing the link between the perceived environmental system (social control and support on behalf of parents and peer influence), personality system (values, expectations, beliefs and attitudes) and behavioural system (health-compromising and health-enhancing behaviours). [2]. Studies show that family environments with their living standards and habitual practices influence children’s lifestyle choices, including their diet preferences, physical activity, habits of personal hygiene, and their mental health [3, 4, 5]. Families could ensure healthy lives, being a supportive and safe space for children’s positive development and providing protection against the negative effects of school stress, peers’ disregard and unhealthy behaviours [6, 7]. Insecure attachment, emotional coldness and authoritative parenting are also associated with decreased mental health and wellbeing of children [8]. Family could be also a conflict-ridden environment with parents’ use of cigarettes, alcohol and even psychoactive substances and could act as a risk factor for health behaviours of children, inducing young people’s stress, depression, behavioural disorders and even suicidal symptoms [9].

According to the latest data from the international Health Behaviour in School-Aged Children survey, round 2018, Bulgarian adolescents lead the ranking on cigarette smoking, alcohol use, drunkenness and early start of sexual life among the countries participating in HBSC-2018 [10]. The main aims of the paper are to highlight the relationships between health risk behaviours (HRBs) of Bulgarian adolescents and family-related characteristics such as *structure of family and its material status, family support, communication with parents, parental monitoring and school-related parental support* and to discuss the key areas for policy intervention concerning young people’s health in Bulgaria, with a focus on the parent support gradient as a sustainable protective factor for adolescent health and wellbeing. In regard to the association between family environment and Bulgarian adolescents’ HRBs, the analysis will focus on the interaction between family affluence and family support. In the second part of the paper we present the main national prevention programs and discuss the parental involvement component in them.

## Material and methods

### Sampling and procedure

The analysis is based on findings of the international survey *Health Behaviour in School-Aged Children* survey (HBSC), conducted in 2018 in Bulgaria. *Health Behaviour Among School-Aged Children* (HBSC) survey is a WHO collaborative cross-national survey of school-aged children’s health and wellbeing, examined in their social context, conducted every four years in about 50 countries of the European WHO Region and Canada. Data are collected in all participating countries through self-report school-based surveys using a standard methodology described in the HBSC 2017/2018 international study protocol [11]. Each country uses random sampling to select a proportion of adolescents aged 11, 13 and 15 years, aiming to achieve representative samples of about 1550 adolescents in each age group. The Bulgarian 2017/2018 HBSC sample was based on a list of all schools that have classes in grades 5, 7 and 9 – the three grades/school years that most closely match 11-, 13- and 15-year-olds. Data were collected in the schools, following permission from the Ministry of Education and Science of Bulgaria. All students who were present and whose parents had given written consent completed the online questionnaire. The final sample comprised 4548 students, of whom 48.4% were male and 51.6% were female. The students were almost equally distributed within the three age groups – 36.7% are 11 years old, 30.1% are 15 years old and 33.4% are 15 years old.

### Statistical analysis

We use descriptive analysis of the prevalence of HRBs of Bulgarian adolescents (current cigarette smoking, regular alcohol consumption, sexual debut and current cannabis use) and logistic regression models in which we control for demographics and family-related characteristics such as structure of family, family affluence scale (FAS – III), family support, communication with mother/father, parental monitoring and school-related parental support. We extend the multivariate analysis, modeling interaction effects between family support and family affluence in order to explore further the association between family support, FAS and children’s HRBs.

### Dependent variables

The dependent variables used in the regression analysis comprise four HRBs that are common in adolescence: current cigarette smoking, regular alcohol consumption, sexual debut and current cannabis use. Within the present study they are constructed as binary variables, reflecting the presence or absence of certain HRB. All scales used in the analyses have been validated and showed high reliability [12].

Adolescents’ current cigarette smoking is measured by a 4-point scale, including the categories ‘every day’, ‘at least once a week’, ‘less than once a week’, and ‘I do not smoke’. The first two categories were combined in the group of students who regularly smoke cigarettes, and the second two categories formed the group of those who do not smoke.

Current alcohol consumption of any of several types of alcohol – beer, wine, spirits/liquor and alcopops – is measured by a 5-point scale, including the categories ‘every day’, ‘every week’, ‘every month’, ‘rarely’ and ‘never’. Daily and weekly drinking were grouped in the category of students who regularly drink alcohol. The other categories were included in the group of non-drinking or rarely drinking adolescents.

The variable on sexual debut assesses the proportion of adolescents who have had sexual intercourse. Only 15-year-old students responded to this question. The created binary variable includes students who have had and students who have not had sexual intercourse.

The variable of current cannabis use assesses the proportion of young people who have used cannabis in the last 30 days. Only 15-year-old students responded to this question. The students who responded that they have never used cannabis in the last month form the category of non-users and the other responses create the groups of students who have used cannabis with differing frequency.

### Independent variables

The present analysis includes as independent variables students’ age, gender and family-related characteristics. The analysis of sexual debut and current cannabis use includes only 15-year-old students.

### Family-related characteristics

The family structure variable examines with whom an adolescent lives all or most of the time, including mother, father, stepmother (father’s partner), stepfather (mother’s partner), living in foster or children’s home, or living with someone/somewhere else. Within the present analysis, the categories that were created comprise the group of students who live with two parents, those who live with one parent and the students living with other relative/somewhere else/in foster care or in children’s home. The distribution of the responses presented in Table 1 reveals that 77.8% from the children live with both parents, 18.9% live in a single parent family and 3.3% live with other relative(s) or in foster care.

**Table 1:** Descriptive statistics of the family characteristics used in the analysis of adolescents’ health risk behaviours (Health Behaviour in School-aged Children (HBSC) – Bulgarian dataset, 2017-2018)

Variable	Categories	Frequency	Percent
	Living with two parents	3537	77.8
**Family structure**	Living with one parent	861	18.9
	Living with other relative/foster care	150	3.3
	Low	498	11.5
**Family affluence scale (FAS-III)**	Medium	3069	70.9
	High	760	17.6
	Low	2859	62.9
**Family support scale**			
	High	1689	37.1
	(Very) easy	3775	84.3
**How easy is it for you to talk to mother about**			
**things that really bother you?**	(Very) difficult	546	12.2
	Don’t have or see mother	155	3.5
	(Very) easy	3285	73.4
**How easy is it for you to talk to father about**			
			
**things that really bother you?**	(Very) difficult	935	20.9
	Don’t have or see father	254	5.7
**Parental monitoring scale - mother**			
	High	1632	35.9
	Low	2916	64.1
	High	1971	43.3
**Parental monitoring scale - father**			
	Low	2577	56.7
**School-related parental support**	High	2791	61.4
	Low	1757	38.6

Family affluence is assessed through the Family Affluence Scale, third revision (FAS III) – a brief assets-based measure including 6 items: number of computers owned by the family, number of cars, number of bathrooms, number of travels/holidays abroad, having own bedroom, and having a dishwasher. The total scale score ranges from 0 to 13, with scores 0-4 representing the category of families with low affluence, the scores 5-9, families with medium affluence, and the scores 10-13, families with high affluence. In the Bulgarian sample 11.5% from the children live in low FAS families, 70.9% live in medium status families and 17.6% are from affluent families.

Family support is a subscale of Multidimensional Scale of Perceived Social Support – MSPSS [13]. It measures the perceived availability of emotional support and help within family. The scale is constructed as a sum of four items assessed through a seven-point scale, ranging from ‘very strongly disagree’ to ‘very strongly agree’. The items of the scale are the following: ‘My family really tries to help me’, ‘I get the emotional help and support I need from my family’, ‘I can talk about my problems with my family’ and ‘My family is willing to help me make decisions’. A cut-off score of 5.5 points was determined to define high family support. In the Bulgarian sample 37.1% of students report high family support. This percentage is the lowest in the international group, compared to other countries participating in HBSC, 2018 [10].

The next two variables reflect the association between students’ HRBs and communication with parents. The question asked separately for mother and father is ‘How easy is it for you to talk to your mother/father about things that really bother you?’. The responses ‘very easy’ and ‘easy’ were combined in the category of easy communication with mother or father. The combined variable of the responses ‘difficult’ and ‘very difficult’ forms the group of students with difficult communication with mother or father. The third category includes those students who ‘don’t have or see mother/father’. The measures are based on the short version of the clear communication scale from the Family Dynamics Measure II (FDM II). Bulgarian students reporting that it is (very) easy for them to talk with mother about things that really bother them are 84.3%. The percentage of those who easily communicate with father is lower: 73.4%.

The association between parental monitoring and adolescents’ HRBs is also tested in the analysis. In the HBSC study parental monitoring reflects parents’ knowledge of their child’s activities, relationships or friends. The measure is based on the instrument developed by Brown et al. [14]. The question asked separately for both parents is ‘How much does your mother / father really know about... who your friends are, how you spend your money, where you are after school, where you go at night, what you do with your free time, what you do on the internet?’. Each item contains four answer options: ‘knows a lot’, ‘knows a little’, ‘doesn’t know anything’, ‘don’t have or see this person’. The valid responses were summed up and the mean was calculated. The scores that are below the mean were combined in the group of students with low communication with mother/ father and the rest created the category of children with high/good communication with mother/father. High parental monitoring on behalf of mother and father is reported by 35.9% and 56.7% of the Bulgarian adolescents.

The last variable used in the analysis is the school-related parental support scale. It is constructed as a sum of five items assessed through a 5-point Likert scale, ranging from ‘very strongly disagree’ to ‘very strongly agree’. The items of the scale are the following: ‘If I have a problem at school, my parents are ready to help’, ‘My parents are willing to come to school to talk to teachers’, ‘My parents encourage me to do well at school’, ‘My parents are interested in what happens to me at school’, ‘My parents are willing to help me with my homework’. The responses on each item were summed up and the mean was calculated. Those students who have a

total score below the mean created the group of children with low school-related parental support and those with scores above the average are combined in the group with high school-related parental support. The Bulgarian students who report high school-related parental monitoring are 61.4%.

## Results

### Prevalence of health risk behaviours among Bulgarian adolescents

In Table 2 the prevalence of HRBs was estimated based on age and gender of children. The results show that smoking and alcohol consumption increases with age. Among the 15-year-old adolescents the prevalence of smoking is higher for girls as compared to boys (19.7% vs. 13.6%). For all age groups the prevalence of drinking is higher among the male students, reaching 21.6% and 18.4% for 15-year-old boys and girls. In regard to sexual debut the results for the 15-year-old students reveal that the prevalence is higher among boys (16.2%) compared to girls (13.6%). The prevalence of cannabis use among 15-year-old boys and girls does not reveal strong gender difference.

**Table 2:** Prevalence of health risk behaviours among Bulgarian adolescents, overall and by age and gender (Health Behaviour in School-aged Children (HBSC) – Bulgarian dataset, 2017-2018).

Health risk behaviour			Overall				Boys	Girls	Boy-to-Girl prevalence ratio

	Age	Total(1)	No. of cases(2)	Boys	Girls	Prevalence	Prevalence	Prevalence	
**Current cigarette smoking**	11 y.o.	1660	118	54	64	7.1	3.3	3.9	0.84
	13 y.o.	1371	152	85	67	11.1	6.2	4.9	1.27
	15 y.o.	1517	506	207	299	33.4	13.6	19.7	0.69
	**Total**	**4548**	**776**	**346**	**430**	**17.1**	**7.6**	**9.5**	**0.80**
	11 y.o.	1660	313	170	143	18.9	10.2	8.6	1.19
	13 y.o.	1371	364	212	152	26.5	15.5	11.1	1.39
	15 y.o.	1517	606	327	279	39.9	21.6	18.4	1.17
**Regular alcohol consumption**	**Total**	**4548**	**1283**	**716**	**726**	**28.2**	**15.7**	**16.0**	**0.99**
**Sexual debut**	15 y.o.	1517	451	245	206	29.7	16.2	13.6	1.19
**Current cannabis use**	15 y.o.	1517	264	133	131	17.4	8.8	8.6	1.02

Legend: (1) All children are included in the total; (2) All children with health risk behaviours are included in the total

### Logistic regression models with main effects on Bulgarian adolescents’ health risk behaviours

The results from the regression models with main effects of all variables reveal significant gender and age differences in some Bulgarian adolescents’ HRBs (Tables 3 and 4, Models 1a-4a). Girls have significantly higher odds of smoking and are less likely to report risky sexual behaviour. The odds of cigarette smoking and regular alcohol consumption increase with age.

**Table 3:** Results of logistic regression models of current cigarette smoking and regular alcohol consumption of Bulgarian adolescents aged 11, 13 and 15 (Health Behaviour in School-aged Children (HBSC) – Bulgarian dataset, 2017-2018).

	Model 1a. Current cigarette smoking - main effects		Model 1b. Current cigarette smoking - interaction effects		Model 2a. Regular alcohol consumption - main effects		Model 2b. Regular alcohol consumption - interaction effects	

	OR	Sig.	OR	Sig.	OR	Sig.	OR	Sig.
**Demographics**:								
**Boy (ref.)**	1		1		1		1	
**Girl**	1.26	*	1.26	**	0.94		0.95	
**11 y.o. (ref.)**	1		1		1		1	
**13 y.o**.	1.44	**	1.45	**	2.75	***	2.75	***
**15 y.o**.	5.89	***	5.90	***	6.40	***	6.42	***
**Structure of family**:								
**Living with both parents (ref.)**	1		1		1		1	
**Living with one parent**	1.60	***	1.60	***	1.34	**	1.34	**
**Living with other relative/foster care**	1.24	*	1.25	*	1.33	**	1.34	**
**Family affluence scale**:								
**Family affluence - low (ref.)**	1				1			
**Family affluence - medium**	1.13				1.11			
**Family affluence – high**	1.12				1.28	*		
**Family support**:								
**Family support - low (ref.)**	1				1			
**Family support – high**	0.65	***			0.79	**		
**Interaction between Family support**								
**and FAS**								
**Fam.support – low & FAS - low**			1.29				1.09	
**Fam.support – low & FAS - medium**			1.45	***			1.18	*
**Fam.support – low & FAS - high**			1.61	**			1.55	**
**Fam.support – high & FAS - low**			0.88				0.88	
**Fam.support – high & FAS - medium (ref.)**			1				1	
**Fam.support – high & FAS - high**			0.79				0.93	
**Communication with parents**:								
**Communication with mother - easy (ref.)**	1		1		1		1	
**Communication with mother – difficult**	1.16		1.16		1.33	**	1.32	*
**No mother**	0.92		0.93		1.20		1.20	
**Communication with father - easy (ref.)**	1		1		1		1	
**Communication with father – difficult**	1.10		1.10		0.95		0.95	
**No father**	1.24		1.24		0.84		0.84	
**Parental monitoring - mother**								
**Low (ref.)**	1		1		1		1	
**High**	1.00		1.00		0.73	***	0.73	***
**Parental monitoring - father**:								
**Low (ref.)**	1		1		1		1	
**High**	0.90		0.89		0.79	**	0.79	**
**School-related parental support**								
**Low (ref.)**	1		1		1		1	
**High**	0.90		0.90		0.84	**	0.84	**
**Constant**	0.07	***	0.05	***	0.22	***	0.20	***
**Log likelihood**	-1675.2		-1674.2		-2295.7		-2294.2	
**Nagelkerke pseudo R-sq**	0.12		0.12		0.12		0.12	
**N of observations**	4127		4127		4190		4190	

Notes: ***: p<0.001, **: p<0.01 *: p<0.05; OR – odds ratios; Sig. – statistical significance; FAS – family affluence scale.

**Table 4:** Results of logistic regression models of sexual debut and current cannabis use of Bulgarian adolescents aged 15 (Health Behaviour in School-aged Children (HBSC) – Bulgarian dataset, 2017-2018).

	Model 3a. Sexual debut - main effects		Model 3b. Sexual debut - interaction effects		Model 4a. Current cannabis use - main effects		Model 4b. Current cannabis use - interaction effects	

	OR	Sig.	OR	Sig.	OR	Sig.	OR	Sig.
**Demographics**:								
**Boy (ref.)**	1		1		1		1	
**Girl**	0.59	***	0.60	***	0.82		0.83	
**Structure of family**:								
**Living with both parents (ref.)**	1		1		1		1	
**Living with one parent**	1.10		1.11		1.74	*	1.74	*
**Living with other relative/ foster care**	1.05		1.05		1.02		1.02	
**Family affluence scale**:								
**Family affluence - low (ref.)**	1				1			
**Family affluence - medium**	0.86				0.85			
**Family affluence – high**	1.00				1.08			
**Family support**:								
**Family support - low (ref.)**	1				1			
**Family support – high**	0.51	***			0.64	*		
**Interaction between Family support and FAS**								
**Fam.support – low & FAS - low**			2.35	***			1.75	**
**Fam.support – low & FAS - medium**			1.65	***			1.30	
**Fam.support – low & FAS - high**			2.19	***			1.97	**
**Fam.support – high & FAS - low**			0.62				0.78	
**Fam.support – high & FAS - medium (ref.)**			1				1	
**Fam.support – high & FAS - high**			0.88				0.82	
**Communication with parents**:								
**Communication with mother - easy (ref.)**	1		1		1		1	
**Communication with mother – difficult**	1.28		1.28		1.20		1.20	
**No mother**	1.67	*	1.66	*	1.01		1.01	
**Communication with father - easy (ref.)**	1		1		1		1	
**Communication with father – difficult**	1.03		1.04		0.94		0.95	
**No father**	0.89		0.89		0.74		0.74	
**Parental monitoring - mother**								
**Low (ref.)**	1		1		1		1	
**High**	1.25	*	1.25	*	0.85		0.85	
**Parental monitoring - father**:								
**Low (ref.)**	1		1		1		1	
**High**	0.99		0.99		0.96		0.96	
**School-related parental support**								
**Low (ref.)**	1		1		1		1	
**High**	0.89		0.89		0.78	*	0.78	*
**Constant**	0.69	*	0.34	***	0.32	***	0.19	***
**Log likelihood**	-832.1		-829.8		-625.9		-624.4	
**Nagelkerke pseudo R-sq**	0.04		0.04		0.03		0.03	
**N of observations**	1411		1411		1438		1438	

Notes: ***: p<0.001, **: p<0.01 *: p<0.05; OR – odds ratios; Sig. – statistical significance; FAS – family affluence scale

Family structure is significantly associated with the studied HRBs, except for sexual debut. Adolescents living with one parent have significantly higher odds of smoking cigarettes, regular alcohol consumption and current cannabis use compared to those living with both parents (reference category).

Family affluence is statistically significant only in the case of drinking alcohol, showing that students from affluent families are more likely to report frequent alcohol consumption.

The models with main effects show also that high family support is associated with significantly lower odds of current cigarette smoking, regular alcohol consumption, early onset of sexual life and current cannabis use.

Difficult communication with mother is associated with significantly higher odds of regular alcohol consumption. Having no mother increases the risk of early sexual debut. Communication with father is not statistically associated with the studied HRBs. High parental monitoring on behalf of mother is negatively associated with the odds of regular alcohol consumption. However, in case of sexual debut the relationship is positive. High monitoring on behalf of father is associated with lower odds of drinking alcohol. The relationship between school-related parental support and regular alcohol consumption or current cannabis use is negative.

### Logistic regression models with interaction effects on Bulgarian adolescents’ health risk behaviours

The models with interaction effect between FAS and family support reveal the following dependencies (Tables 3 and 4, Models 1b-4b). The effects of age and gender do not change, as a direction and magnitude of the differences, compared to the models with main effects. Girls have higher odds of smoking cigarettes and are less likely to report risky sexual behaviour compared to boys. Gender differences in the odds of alcohol consumption and cannabis use are not significant. Increasing age is associated with higher likelihood of cigarette smoking and drinking alcohol.

The effect of family structure does not change either, showing that adolescents living with one parent or in other settings are more likely to report frequent smoking, drinking and cannabis use.

Interestingly, the results from the models including interaction effects between FAS and family support reveal that students who report low family support in different material status families have significantly higher odds of HRBs compared to the reference category – students from medium FAS families with high family support. In regard to the students from high support families, the differences attributed to FAS decrease and become insignificant.

Difficult communication with mother is negatively associated with frequent alcohol consumption, while having no mother is associated with increased likelihood of early sexual debut. High parental monitoring both from mother or father is associated with lower odds of regular alcohol consumption. Similar to model 3a, in the case of sexual debut, higher monitoring on behalf of mother is reduces risky sexual behaviour. Higher school-related parental support has a preventive effect on regular alcohol consumption and current cannabis use.

## Discussion

The multivariate analyses reveal significant gender differences in the risk of smoking and risky sexual behaviour of Bulgarian adolescents. Existing studies show that gender differences in cigarette smoking are ambivalent in the different contexts. Grard et al. [15], for example, reveal that higher prevalence of smoking among girls in European countries is related to gender differences in smoking beliefs, with those related to dating aspects being more common among boys. Sanchez et al. [16] find no difference in the prevalence of recent cigarette smoking between boys and girls in private school–attending youths in a Latin American context. Cui et al. [17] reveal higher prevalence of smoking among male adolescents in Asian countries. The findings from our study are in line with Pop et al. [18], who reveal significant gender-related differences between never-smokers and experimental adolescent smokers in Romania, emphasising the need for gender-tailored smoking prevention programs. In regard to gender differences in sexual debut, uncovered in the present analysis, existing studies show that early onset of sexual life is associated with a higher proportion of adolescents who are potentially at risk for a range of poor reproductive health outcomes, which signals the need for gender-tailored prevention programs focused on young people’s reproductive health [19].

The odds of cigarette smoking and regular alcohol consumption among Bulgarian students increase with age. Many studies find also that alcohol and tobacco use exhibited monotonic increases over adolescence and young adulthood [e.g. 20].

Bulgarian adolescents living with one parent have significantly higher odds of cigarette smoking, regular alcohol consumption and current cannabis use compared to those living with both parents. Recent study conducted by Park and Lee [21] finds also that adolescents living in single-parent families are more vulnerable to the health risks of smoking, drinking, risky sexual behaviour and mental health issues.

Students from more affluent families in Bulgaria are more likely to report frequent alcohol consumption. This result is in line with previous findings based on HBSC, showing that in Bulgaria the association between some of the adolescents’ HRBs and FAS is positive [22].

High family support is associated with significantly lower odds of current cigarette smoking, regular alcohol consumption, sexual debut and current cannabis use among Bulgarian students. Existing studies found also a positive relationship between family (parental) support and adolescents’ mental and physical health and health behaviours [23]. However, in our study the interaction between FAS and family support shows that high family support alleviates the negative effect of socio-economic inequalities between families and has a preventive effect on young peoples’ HRBs.

The present study affirms the role of family communication as a protective factor of adolescents’ health and well-being. The results are also in line with previous findings, revealing stronger protective effects of communication with mother [24, 25, 26]. Parental monitoring and school-related parental support are also significantly associated with adolescents’ HRBs. However, this relationship is ambivalent. DiClemente et al. [27] find that adolescents perceiving less parental monitoring were more likely to have risky sexual behaviours, a history of marijuana use, and to use marijuana more often in the past 30 days, to have a history of alcohol use and greater alcohol consumption in the past 30 days. Bulgarian data seem to be opposite in the case of sexual debut, revealing that higher parental monitoring (mother) is positively associated with risky sexual behaviour. Studies find that the protective effect of parental monitoring on risky sexual behaviour is moderated by gender. Findings from a review study done by Kincaid et al. [28] show that parental ‘monitoring may be more protective against sexual risk behaviour for boys than girls, whereas parental warmth and emotional connection may be an especially salient factor for girls’. In the present study higher parental monitoring may be associated with an authoritative and controlling parenting style that could have an ambivalent effect on adolescents’ risky sexual behaviour.

### Family support programs and interventions

Evidence-based conclusions that families have been a protective factor against adolescents’ HRBs make development of family support programs of crucial importance for health policy and practice. Family support includes a wide range of interventions that may happen in different settings (home, school, community), with a focus on different problems (child neglect, parents’ conflicts, children’s behavioural disorders, etc.), involving professionals and/or (only) family members, with educational and/or therapeutic aims. As a diverse concept ‘family support’ aims at increasing parents’ capacities and skills in order to promote children’s physical and psychosocial wellbeing [29, 30]. As stated in the UNICEF report on families, family policies and developmental sustainable goals, there is no ‘silver bullet’ in family policy or program design, but aspects of different policies are shown to be effective in different settings when designed for a specific purpose [31].

At the policy level, parents as a target group and their involvement have been stipulated in national legal documents concerning children’s health, education and wellbeing (Preschool and School Education Act, Health Act and Social Services Act, National Strategy for Combatting Drugs Use 2020-2024). However, mainstreaming the family dimension is lacking in every policy, and no comprehensive vision for family support policy and service provision for families exists.

At a practical level, a network of state-run units function at national and municipal levels which tackle the problems of drug use and alcohol and nicotine addictions, such as the Central Commission for Combatting Anti-Social Behaviour of Minors and Its Local Branches, local centres for drug prevention and information, and councils on prevention of drug use in municipalities. In these programs and projects on drug prevention, parents are targeted as a group for interventions, and training seminars, family therapy and individual counselling are provided, with a focus on stimulating positive parenting, effective communication between parents and teenagers, building skills for emotional integrity in family, and so on. School for Parents and Workshops for Parents are the two most attractive programs where family professionals equip parents with knowledge and skills on how to communicate with their children on difficult topics, including addictions, violence and anti-social behaviour, how to prevent or overcome conflicts, and more. Additionally, health promotion information campaigns with adolescents and parents are organised as a component of the prevention activities by the local commissions for combatting anti-social behaviour of minors in cooperation with the local Health Inspection units.

Some NGOs such as Solidarnost1http://www.solidarnost-bg.org/en/rehabilitation-program/work-with-families/.Accessed [14.07.2021]. and Mothers Against Drugs also have family support programs as part of their rehabilitation and reintegration programs for addicts. The NGOs organise parental support groups and individual counselling for parents to help them cope with behaviours and dysfunction of their children, to reduce family conflicts and enable acceptance of changes in their addicted children.

In spite of the above-mentioned programs, services for parents are limited and still there are unmet needs for affordable counselling services for parents on issues of addiction, including telephone and online consultations. The need for more family support services is one of the experts’ recommendations concerning adolescent health policies in a recent national report [32]. Additionally, the absence of evaluation and monitoring of programs makes difficult to assess their efficacy for better family functioning and for positive child development.

The Schools for Health in Europe (SHE) approach [33, 34] sets up a holistic view of adolescents’ health, giving students knowledge and skills that enable them to make healthy choices and that stimulate their positive development. Schools are viewed as not only educational but relational settings to build meaningful and cooperative relations, to encourage positive adolescent behaviours, and to reduce risks and negative outcomes from unhealthy behaviours [35]. In the SHE perspective, schools develop collaborations and partnerships with parents in order to activate their participation in lessons, workshops and other activities that strengthen their skills in health promotion. National data show that in today’s schools teachers and parents do not collaborate effectively; teachers are predominantly engaged with a small number of parents of children with low achievements and problematic behaviour, and the potential of the rest of the parents to cooperate with the school has been neglected [36]. Thus, using and multiplying parents’ capacities in school activities conducive to health promotion, is a valuable way to strengthen life skills education for young people.

## Conclusion

The present study reveals that high family support, good communication in the family, parental engagements and school-related parental support have a preventive effect on the HRBs of young people in Bulgaria. This evidence-based approach delineating family support as a crucial determinant of adolescents’ health behaviours, reducing the effect of material status inequalities, provides solid arguments for increasing family support programs as a means to promote healthy lifestyles among young people. The results from the statistical analysis and the overview of intervention programs targeting adolescent health demonstrate that the family support component has been underestimated in research and practice. Child-Parent-Teacher communication is underestimated in the national context as an effective means to support adolescents’ health and wellbeing. More active parents’ inclusion in the extra-class activities during their childrens’ adolescence is needed in order to provide better partnerships between parents, teachers and students concerning health promotion. Development of more services for parent counselling is of crucial importance for effective promotion of healthy lifestyles of young people in Bulgaria.

### Кey points

- Family environment plays a crucial role in adolescents’ HRBs – family support significantly reduces the negative effect of socio-economic inequalities between families and has a protective effect on young people’s health and wellbeing.
- Good communication in families and school-related parental monitoring are positive factors for prevention of adolescents’ unhealthy behaviours.

• Family support programs are needed for healthier family functioning as a means to reduce adolescent health risk behaviours and to alleviate the negative effect of material status inequalities.

- Using and multiplying parents’ capacities in school activities related to health promotion is a valuable way to strengthen life skills education for young people.
